# Supplementary material for: Genome-Wide Identification of the Maize Chitinase Gene Family and Analysis of Its Response to Biotic and Abiotic Stresses
Source: Genes (Basel). 2024 Oct 15;15(10):1327. doi: 10.3390/genes15101327 (PMC11507598; doi:10.3390/genes15101327)
Supplement: Supplementary file 1 [file genes-15-01327-s001.zip › Supplementary Table S1.pdf]

Supplementary Table S1: URLs and access times for all sites in "Materials and methods"

Serial number	Website	Web link	Query time
1	Phytozome database	https://phytozome-next.jgi.doe.gov/info/Zmays_Zm_B73_REFERENCE_NAM_5_0_55	accessed 10 April 2024
2	Arabidopsis genome database	https://www.arabidopsis.org/	accessed 10 April 2024
3	Pfam database	https://www.ebi.ac.uk/interpro/entry/pfam/	accessed 10 April 2024
4	HMMERv3.3.2 Website	https://www.ebi.ac.uk/Tools/hmmer/	accessed 10 April 2024
5	SMART	http://smart.embl-heidelberg.de	accessed 11 April 2024
6	CDD	http://www.ncbi.nlm.nih.gov/Structure/cdd/wrpsb.cgi	accessed 11 April 2024
7	ExPASy	https://web.expasy.org/protparam/	accessed 13 April 2024
8	Cell-PLoc	http://www.csbio.sjtu.edu.cn/bioinf/Cell-PLoc/	accessed 6 October 2024
9	WoLF PSORT	https://wolfpsort.hgc.jp/	accessed 13 April 2024
10	NCBI database	https://www.ncbi.nlm.nih.gov/cdd	accessed 13 April 2024
11	MEME	http://meme-suite.org/	accessed 13 April 2024
12	Evolview	https://www.evolgenius.info/evolview	accessed 17 April 2024
13	PlantCare	https://bioinformatics.psb.ugent.be/webtools/plantcare/html/	accessed 25 April 2024
14	ShinyGO v0.741: Gene Ontology Enrichment Analysis + more	http://bioinformatics.sdstate.edu/go74/	accessed 3 May 2024
15	Microbiosense	https://www.bioinformatics.com.cn/	accessed 5 May 2024
16	FastQC	https://github.com/s-andrews/FastQC	accessed 13 May 2024
