# Supplementary material for: Genome-Wide Identification of the Maize Chitinase Gene Family and Analysis of Its Response to Biotic and Abiotic Stresses
Source: Genes (Basel). 2024 Oct 15;15(10):1327. doi: 10.3390/genes15101327 (PMC11507598; doi:10.3390/genes15101327)
Supplement: Supplementary file 1 [file genes-15-01327-s001.zip › Supplementary Table S2.pdf]

Supplementary Table S2: Physiochemical properties of the proteins encoded by 43 maize chitinase gene family members.

Gene Name	Sequence ID	CDS Size (bp)	Number of Amino Acids (aa)	Molecular Weight (kD)	pI	Instability Index	Aliphatic Index	Grand Average of Hydropathicity
ZmChi1	Zm00001eb002620	783	261	28.24	8.99	40.3	57.74	-0.356
ZmChi2	Zm00001eb007850	594	198	21.71	5.92	51.05	91.72	0.083
ZmChi3	Zm00001eb008880	1797	599	66.13	6.47	45.9	84.71	-0.197
ZmChi4	Zm00001eb022500	630	210	22.59	4.79	32.21	62.86	-0.311
ZmChi5	Zm00001eb047940	924	308	33.42	4.11	36.5	84.94	0.074
ZmChi6	Zm00001eb078720	834	278	28.99	7.86	30.89	58.02	-0.128
ZmChi7	Zm00001eb078730	840	280	29.21	8.44	30.14	57.68	-0.216
ZmChi8	Zm00001eb078740	681	227	23.7	7.57	37.31	69.34	-0.019
ZmChi9	Zm00001eb147120	933	311	32.4	9.12	40.59	77.27	0.055
ZmChi10	Zm00001eb147140	897	299	31.96	5.92	39.99	85.22	-0.005
ZmChi11	Zm00001eb147150	882	294	31.12	4.93	34.45	82.01	-0.086
ZmChi12	Zm00001eb147160	921	307	33.34	8.54	38.14	82.02	-0.172
ZmChi13	Zm00001eb157820	885	295	30.29	4.06	31.04	83.39	0.053
ZmChi14	Zm00001eb167340	903	301	32.54	8.69	35.86	84.42	-0.095
ZmChi15	Zm00001eb168350	858	286	31.13	4.97	23.99	82.83	-0.008
ZmChi16	Zm00001eb169950	1362	454	48.39	5.08	41.71	86.01	0.08
ZmChi17	Zm00001eb174340	2172	724	77.31	8.17	48.07	80.08	-0.123
ZmChi18	Zm00001eb228500	846	282	29.59	5.1	33.84	61.88	-0.121
ZmChi19	Zm00001eb228510	579	193	19.82	9.46	67.31	41.76	-0.518
ZmChi20	Zm00001eb246640	813	271	28.56	5.14	26.1	66.79	-0.064
ZmChi21	Zm00001eb250900	858	286	31.29	4.99	28.44	81.15	-0.044
ZmChi22	Zm00001eb266300	1635	545	60.57	5.78	42.93	87.52	-0.107
ZmChi23	Zm00001eb270440	288	96	10.56	9.1	65.02	79.38	0.153
ZmChi24	Zm00001eb270450	285	95	11.03	4.17	49.82	53.58	-0.659
ZmChi25	Zm00001eb272050	1137	379	40.38	4.82	43.94	60.9	-0.174
ZmChi26	Zm00001eb272090	783	261	27.34	10.35	58.83	75.25	-0.213
ZmChi27	Zm00001eb283260	945	315	34.18	6.95	40.68	78.03	-0.234
ZmChi28	Zm00001eb283280	1104	368	40.35	5.72	40.54	79.54	-0.187
ZmChi29	Zm00001eb288150	1587	529	58.56	5.94	38	91.3	-0.088
ZmChi30	Zm00001eb301490	921	307	32.99	9.21	34.58	84.36	-0.028
ZmChi31	Zm00001eb301500	936	312	34.24	7.11	42.88	76.38	-0.181
ZmChi32	Zm00001eb305260	891	297	31.14	4.65	33.03	81.85	-0.023
ZmChi33	Zm00001eb305270	891	297	31.17	4.65	33.71	80.2	-0.052
ZmChi34	Zm00001eb305280	891	297	31.22	4.74	32.84	81.85	-0.044
ZmChi35	Zm00001eb317090	984	328	36.35	8.16	29.29	63.75	-0.319
ZmChi36	Zm00001eb325280	1734	578	62.89	5.15	30.86	85.95	-0.083
ZmChi37	Zm00001eb332350	855	285	31.27	6.97	46.7	64.18	-0.281
ZmChi38	Zm00001eb340820	804	268	29.46	8.09	35.09	56.53	-0.271
ZmChi39	Zm00001eb346860	1071	357	37.92	8.04	36.7	60.81	-0.215

ZmChi40	Zm00001eb354540	933	311	33.78	5.97	38.35	67.56	-0.207
ZmChi41	Zm00001eb358410	1290	430	48.27	8.42	44.07	95	-0.202
ZmChi42	Zm00001eb420850	1452	484	50.61	8.88	40.28	86.9	0.212
ZmChi43	Zm00001eb425600	843	281	29.13	8.92	28.11	57.08	-0.158
