# Supplementary material for: Genome-Wide Identification of the Maize Chitinase Gene Family and Analysis of Its Response to Biotic and Abiotic Stresses
Source: Genes (Basel). 2024 Oct 15;15(10):1327. doi: 10.3390/genes15101327 (PMC11507598; doi:10.3390/genes15101327)
Supplement: Supplementary file 1 [file genes-15-01327-s001.zip › Supplementary Table S3.pdf]

Supplementary Table S3: Subcellular prediction results.

Gene Name	WoLF PSORT Web site search	Cell-PLoc Web site search
ZmChi1	chlo: 5, extr: 5, vacu: 2, cyto: 1, plas: 1	Extracell.
ZmChi2	extr: 4, cyto: 2.5, chlo: 2, vacu: 2, cyto_nucl: 2, mito: 1, E.R.: 1, pero: 1	Chloroplast. Nucleus. Vacuole.
ZmChi3	chlo: 14	Cell wall. Chloroplast.
ZmChi4	extr: 8, chlo: 3, nucl: 1, cyto: 1, mito: 1	Extracell.
ZmChi5	chlo: 9, extr: 2, cyto: 1.5, cyto_nucl: 1.5, mito: 1	Cell membrane. Cell wall.
ZmChi6	chlo: 14	Vacuole.
ZmChi7	extr: 13, vacu: 1	Extracell. Vacuole.
ZmChi8	extr: 7, mito: 3, E.R.: 2.5, E.R._plas: 2.5, plas: 1.5	Extracell.
ZmChi9	chlo: 14	Vacuole.
ZmChi10	chlo: 7, pero: 5, cyto: 1.5, cyto_nucl: 1.5	Extracell.
ZmChi11	chlo: 5, E.R.: 3, plas: 2, cyto: 1.5, cyto_nucl: 1.5, vacu: 1, pero: 1	Vacuole.
ZmChi12	cyto: 4, chlo: 2, plas: 2, E.R.: 2, nucl: 1, mito: 1, vacu: 1, pero: 1	Extracell.
ZmChi13	extr: 6, mito: 3, vacu: 2, E.R._plas: 2, plas: 1.5, E.R.: 1.5	Extracell.
ZmChi14	vacu: 6, extr: 5, chlo: 1, nucl: 1, mito: 1	Extracell.
ZmChi15	cyto: 6, chlo: 5, mito: 2, cysk_nucl: 1	Cell wall.
ZmChi16	chlo: 5, plas: 3.5, E.R._plas: 3.5, E.R.: 2.5, extr: 2, pero: 1	Cell wall.
ZmChi17	plas: 7, E.R.: 4, chlo: 1, mito: 1, pero: 1	Chloroplast.
ZmChi18	chlo: 7, extr: 6, vacu: 1	Vacuole.
ZmChi19	extr: 10, chlo: 2, vacu: 2	Cell wall. Vacuole.
ZmChi20	chlo: 8, extr: 4, vacu: 2	Vacuole.
ZmChi21	cyto: 5, chlo: 3, mito: 2, extr: 2, nucl: 1.5, cysk_nucl: 1.5	Cell membrane. Cell wall.
ZmChi22	chlo: 5, vacu: 5, mito: 2, nucl: 1, E.R.: 1	Cell wall. Chloroplast.
ZmChi23	extr: 8, vacu: 4, chlo: 2	Cell membrane. Cell wall. Extracell. Vacuole.
ZmChi24	mito: 8, chlo: 4, nucl: 2	Cell wall.
ZmChi25	E.R.: 4.5, E.R._plas: 3.5, mito: 3, cyto: 2.5, cyto_pero: 2, plas: 1.5, chlo: 1, nucl: 1	Vacuole.

ZmChi26	extr: 7, vacu: 7	Vacuole.
ZmChi27	chlo: 12, nucl: 1, mito: 1	Extracell.
ZmChi28	chlo: 6, plas: 2, vacu: 2, E.R.: 2, cyto: 1, extr: 1	Extracell.
ZmChi29	chlo: 4, E.R.: 3, mito: 2, vacu: 2, nucl: 1.5, cyto_nucl: 1.5, plas: 1	Chloroplast.
ZmChi30	extr: 6, chlo: 3, vacu: 2, nucl: 1, mito: 1, golg: 1	Extracell.
ZmChi31	mito: 6, chlo: 2, vacu: 2, E.R.: 2, cyto: 1, golg: 1	Extracell.
ZmChi32	extr: 8, mito: 3, E.R._plas: 2, plas: 1.5, E.R.: 1.5	Vacuole.
ZmChi33	extr: 8, mito: 2, E.R._plas: 2, plas: 1.5, E.R.: 1.5, vacu: 1	Vacuole.
ZmChi34	extr: 8, mito: 2, E.R._plas: 2, plas: 1.5, E.R.: 1.5, vacu: 1	Vacuole.
ZmChi35	extr: 4, mito: 3, vacu: 2, cyto_nucl: 2, nucl: 1.5, cyto: 1.5, chlo: 1, plas: 1	Vacuole.
ZmChi36	extr: 9, E.R.: 3, chlo: 1, mito: 1	Cell wall. Chloroplast.
ZmChi37	chlo: 10, extr: 2, vacu: 2	Extracell. Vacuole.
ZmChi38	chlo: 9, extr: 3, vacu: 2	Extracell.
ZmChi39	chlo: 5, cyto: 3.5, cyto_nucl: 3, extr: 2, nucl: 1.5, plas: 1, E.R.: 1	Vacuole.
ZmChi40	chlo: 13, extr: 1	Extracell. Vacuole.
ZmChi41	plas: 4, mito: 3, nucl: 2, golg: 2, cyto: 1, vacu: 1, pero: 1	Cell wall.
ZmChi42	chlo: 3, plas: 3, E.R.: 3, extr: 2, vacu: 2, mito: 1	Cell wall.
ZmChi43	extr: 10, vacu: 2, chlo: 1, golg: 1	Vacuole.
