# Supplementary material for: Genome-Wide Identification of the Maize Chitinase Gene Family and Analysis of Its Response to Biotic and Abiotic Stresses
Source: Genes (Basel). 2024 Oct 15;15(10):1327. doi: 10.3390/genes15101327 (PMC11507598; doi:10.3390/genes15101327)
Supplement: Supplementary file 1 [file genes-15-01327-s001.zip › Supplementary Table S4.pdf]

Supplementary Table S4: Analysis of genetic replication events of the chitinase family in Zea mays.

Duplicated genes		Ka	Ks	Ka/Ks	Date (MY)	Duplication Type
ZmChi6	ZmChi7	0.154	0.239	0.644	18.36	Tandem replications
ZmChi7	ZmChi8	0.18	0.277	0.65	21.34	Tandem replications
ZmChi9	ZmChi10	0.318	0.371	0.855	28.57	Tandem replications
ZmChi10	ZmChi11	0.138	0.337	0.411	25.89	Tandem replications
ZmChi11	ZmChi12	0.114	0.324	0.352	24.95	Tandem replications
ZmChi18	ZmChi19	0.431	0.759	0.568	58.36	Tandem replications
ZmChi30	ZmChi31	0.347	0.424	0.818	32.61	Tandem replications
ZmChi32	ZmChi33	0.006	0.086	0.07	6.63	Tandem replications
ZmChi33	ZmChi34	0.008	0.051	0.146	3.95	Tandem replications
ZmChi1	ZmChi18	0.385	0.7	0.55	53.83	Fragment replications
ZmChi43	ZmChi6	0.208	0.231	0.9	17.74	Fragment replications
ZmChi43	ZmChi20	0.322	0.39	0.826	30.02	Fragment replications
ZmChi6	ZmChi20	0.307	0.39	0.787	30.02	Fragment replications
ZmChi18	ZmChi25	0.28	0.412	0.678	31.72	Fragment replications
ZmChi37	ZmChi40	0.271	0.601	0.451	46.2	Fragment replications
