# Supplementary material for: Genome-Wide Identification of the Maize Chitinase Gene Family and Analysis of Its Response to Biotic and Abiotic Stresses
Source: Genes (Basel). 2024 Oct 15;15(10):1327. doi: 10.3390/genes15101327 (PMC11507598; doi:10.3390/genes15101327)
Supplement: Supplementary file 1 [file genes-15-01327-s001.zip › Supplementary Table S5.pdf]

Supplementary Table S5: Homology of chitinase proteins in maize, Arabidopsis, and rice.

orthologous gene pairs	
ZmChi1	Os03g04060
ZmChi4	Os10g39680
ZmChi5	Os10g28050
ZmChi9	Os01g64110
ZmChi12	Os01g64100
ZmChi13	Os01g47070
ZmChi16	Os11g27400
ZmChi20	Os02g39330
ZmChi31	Os06g25010
ZmChi35	Os09g32080
ZmChi39	Os05g33130
ZmChi40	Os05g04690
ZmChi42	Os04g30770

paralogous homologous gene pairs			
ZmChi21	ZmChi15	At2g43620	At2g43610
ZmChi24	ZmChi37	At4g01700	At1g02360
ZmChi26	ZmChi18	At4g19740	At4g19730
ZmChi28	ZmChi27	At4g19760	At4g19750
ZmChi29	ZnChi22	At4g19820	At4g19810
ZmChi34	ZmChi32	At1g02360	At4g01700
ZmChi36	ZmChi3	Os04g41680	Os04g41620
ZmChi43	ZmChi7	Os05g15850	Os05g15770
ZmChi8	ZmChi6	Os05g15920	Os05g15880
		Os06g51060	Os06g51050
		Os07g23850	Os04g27980
		Os10g28120	Os10g28080
		Os11g47510	Os11g47500
		Os11g47520	Os08g40680
		Os11g47570	Os11g47560
		Os11g47580	Os08g40740
		Os11g47610	Os11g47590
