# Supplementary material for: Genome-Wide Identification of the Maize Chitinase Gene Family and Analysis of Its Response to Biotic and Abiotic Stresses
Source: Genes (Basel). 2024 Oct 15;15(10):1327. doi: 10.3390/genes15101327 (PMC11507598; doi:10.3390/genes15101327)
Supplement: Supplementary file 1 [file genes-15-01327-s001.zip › Supplementary Table S6.pdf]

Supplementary Table S6: Motif data for chitinase proteins in Zea mays.

Motif	Sequence	Number of Amino Acids	Pfam Annotation
Motif1	YYGRGPJQJSWNYNYGPAGQAJGFDGLGBPDLVAQDPVVAFKTAL WFWMT	50	GH19
Motif2	VPADDLYSQVLPLIKKSPKYGGIMJWSRYYDDQTGYSSAVK	41	GH18
Motif3	AGSIAVYWGQNGGEGTLAETCATGNYRFVIJAFLSVFGNGQ	41	GH18
Motif4	ALNTGLFDYVWVQFYNNPPCQ	21	\
Motif5	SRPLGDAVLDGIDFDIEGGTAKHWDDLARYLK	32	GH18
Motif6	PSSGGCTGLSGDIKSCQSSGVKVLLSIGGGAGSYGLSS	38	GH18
Motif7	CPAKGFYTYDAFJAAAAAFPG	21	GH19
Motif8	PAAVNDRVGYYKRYCDQFGVDPGNNLTC	28	GH19
Motif9	VASIVAEYLWNNFLGGRSS	19	GH18
Motif10	AAQGAGCPPCLCCSKFGYCGSTSDYCG	27	GH18
Motif11	FGTTGTEDTRKREVAAFLAQVSHETTGGW	29	GH19
Motif12	RPPGFGATTNIINGALECGGG	21	GH18
