# Supplementary material for: Genome-Wide Identification of the Maize Chitinase Gene Family and Analysis of Its Response to Biotic and Abiotic Stresses
Source: Genes (Basel). 2024 Oct 15;15(10):1327. doi: 10.3390/genes15101327 (PMC11507598; doi:10.3390/genes15101327)
Supplement: Supplementary file 1 [file genes-15-01327-s001.zip › Supplementary Table S7.pdf]

Supplementary Table S7: Tissue specificity of maize chitinases and potential candidate genes under biotic and abiotic stresses.

**Tissue-specific potential candidate genes**	24H_Germinating Seed	ZmChi28	ZmChi31	ZmChi43	
	6DAS_GH_Primary Root	ZmChi13	ZmChi29	ZmChi35	
	V3_Stem and SAM	ZmChi1	ZmChi13	ZmChi35	ZmChi7
	V5_Tip of stage-2 Leaf	ZmChi1			
	V9_Immature Leaves	ZmChi29	ZmChi35		
	V9_Thirteenth Leaf	ZmChi1	ZmChi22	ZmChi35	
	V9_Eleventh Leaf	ZmChi1	ZmChi35		
	V9_Eighth Leaf	ZmChi1	ZmChi13		
	VT_Thirteenth Leaf	ZmChi1	ZmChi13		
	R2_Thirteenth Leaf	ZmChi1	ZmChi13	ZmChi26	
	10DAP_Whole seed	ZmChi1	ZmChi35		
	12DAP_Whole seed	ZmChi1	ZmChi35		
	14DAP_Whole seed	ZmChi35			
	16DAP_Whole seed	ZmChi43			
	16DAP_Endosperm	ZmChi43			

**Potential candidate genes for biotic versus abiotic stresses**	Temperature	ZmChi5	ZmChi7	ZmChi9	ZmChi13	ZmChi15	ZmChi26	ZmChi28	ZmChi30	ZmChi31	ZmChi43			
	Waterflooding	ZmChi21	ZmChi28	ZmChi30	ZmChi31	ZmChi39								
	Drought	ZmChi5	ZmChi7	ZmChi18	ZmChi19	ZmChi31	ZmChi37	ZmChi43						
	Salt	ZmChi7	ZmChi8	ZmChi13	ZmChi14	ZmChi15	ZmChi26	ZmChi28	ZmChi30	ZmChi31	ZmChi39	ZmChi43		
	fluctuations in the light	ZmChi9	ZmChi14	ZmChi15	ZmChi26	ZmChi31	ZmChi36							
	Smut disease	ZmChi5	ZmChi6	ZmChi7	ZmChi13	ZmChi14	ZmChi15	ZmChi18	ZmChi26	ZmChi28	ZmChi30	ZmChi39	ZmChi43	
	Gibberella stalk rot	ZmChi3	ZmChi4	ZmChi5	ZmChi6	ZmChi8	ZmChi13	ZmChi15	ZmChi16	ZmChi20	ZmChi21	ZmChi26	ZmChi28	ZmChi30
	Gray leaf spot	ZmChi9	ZmChi13	ZmChi15	ZmChi18	ZmChi19	ZmChi30	ZmChi37						
	Spodoptera exigua	ZmChi7	ZmChi13	ZmChi15	ZmChi20	ZmChi26	ZmChi28	ZmChi29	ZmChi31					
	Ostrinia furnacalis	ZmChi7	ZmChi14	ZmChi18	ZmChi19	ZmChi25	ZmChi26	ZmChi28	ZmChi31					
	Aphid	ZmChi15	ZmChi20	ZmChi31										
